# Identification of retinol dehydrogenase 10 as a shared biomarker for metabolic dysfunction-associated steatotic liver disease and type 2 diabetes mellitus

**DOI:** 10.3389/fphar.2025.1521416

**Published:** 2025-01-24

**Authors:** Fangyu Li, Rui Li, Hongjun Deng

**Affiliations:** ^1^ Department of Endocrinology, Union Hospital, Tongji Medical College, Huazhong University of Science and Technology, Wuhan, China; ^2^ Department of Rehabilitation Medicine, Union Hospital, Tongji Medical College, Huazhong University of Science and Technology, Wuhan, China

**Keywords:** retinol dehydrogenase10, MASLD, T2DM, bioinformatics, biomarker

## Abstract

**Background:**

Metabolic dysfunction-associated steatotic liver disease (MASLD) is an independent risk factor for type 2 diabetes mellitus (T2DM), and its early identification and intervention offer opportunities for reversing diabetes mellitus.

**Methods:**

In this study, we identified biomarkers for the MASLD dataset (GSE33814, GSE48452) and the T2DM dataset (GSE76895 and GSE89120) by bioinformatics analysis. Next, we constructed weighted gene co-expression network (WGCNA) for disease module analysis to screen out shared genes strongly associated with diseases. We also analyzed the enriched pathways of shared genes using Gene Ontology (GO) and Kyoto Encyclopedia of Genes and Genomes (KEGG) pathways. Next, hub gene validation was performed using the least absolute shrinkage and selection operator (LASSO) and receiver operating characteristic (ROC) curves. Finally, we used RT-qPCR, immunofluorescence, Western blotting and Elisa to validate hub gene expression in MASLD and T2DM mouse models.

**Results:**

This analysis identified 20 genes shared by MASLD and T2DM that were enriched in the bile secretion, phototransduction, cancer, carbohydrate digestion and absorption, cholesterol/glycerol metabolism, and retinol metabolism. The LASSO algorithm and ROC curve identified *Retinol Dehydrogenase 10* (*RDH10)* as the best diagnostic gene for MASLD and T2DM. Immunofluorescence and Western blot showed that RDH10 expression was reduced in the liver and pancreatic islets of MASLD and T2DM model mice. Similarly, serum levels of RDH10 were significantly lower in MASLD and T2DM model mice and humans than in controls.

**Conclusion:**

Our study suggests that RDH10 is a common diagnostic marker for MASLD and T2DM and provides new research directions for the prevention and treatment of MASLD and T2DM.

## 1 Introduction

Metabolic dysfunction-associated steatotic liver disease (MASLD), previously known as non-alcoholic fatty liver disease (NAFLD), is defined as fatty liver disease in the presence of at least one cardiometabolic risk factor (overweight/obesity/central obesity, impaired glucose regulation or diabetes, high blood pressure, hypertriglyceridaemia, and reduced HDL-cholesterol) and in the absence of harmful alcohol intake (Rinella et al., 2023; Song et al., 2024). MASLD is a prevalent global health concern, with an increasing incidence rate on an annual basis. It is one of the principal causes of chronic liver disease, affecting approximately one-third of the adult population in Western countries at a rate commensurate with the global epidemics of obesity and diabetes (Riazi et al., 2022; Younossi, 2019). Insulin resistance and chronic inflammation are significant contributing factors to the development of MASLD (Targher et al., 2018). Consequently, MASLD frequently coexists with a range of metabolic disorders, including obesity, type 2 diabetes mellitus (T2DM), and hypertension (Younossi et al., 2018; Lonardo et al., 2018).

A number of studies have identified a complex bidirectional relationship between MASLD and T2DM (Younossi et al., 2016; Portillo-Sanchez et al., 2015; Birkenfeld and Shulman, 2014). The presence of other common metabolic risk factors notwithstanding, patients with MASLD exhibit a two-fold increased risk of developing T2DM (Targher et al., 2021). Furthermore, the risk of developing T2DM correlates with the severity of MASLD (Targher et al., 2018). The progression of MASLD leads to an increase in the secretion of several hepatokines, which exacerbates insulin resistance and, together with the resulting increases in hyperglycemia, serum free fatty acids (FFAs), and inflammation, induce glycolipotoxicity and deterioration of β-cell function (Pal et al., 2012; El Ouaamari et al., 2016; López-Bermudo et al., 2022; Scoditti et al., 2024). In addition, MASLD in patients with T2DM combined with MASLD progresses more rapidly to MASH, hepatic fibrosis, and cirrhosis, thereby increasing the risk of adverse intra- and extrahepatic clinical outcomes (Stepanova et al., 2013; En et al., 2023). Therefore, early identification and intervention of MASLD is crucial for the prevention and treatment of T2DM.

The aim of this study is to screen for MASLD as well as T2DM in populations at high metabolic risk, to facilitate early detection and treatment in order to reduce the burden of T2DM-MASLD. There is considerable heterogeneity in the prevalence of MASLD when different diagnostic methods are employed (Younossi et al., 2016; Younossi et al., 2019). Therefore, there is an urgent need to find an early marker to recognize both diseases. At present, the pharmacological treatment of MASLD has not been approved by international regulatory authorities. A range of drug classes used for the treatment of hyperglycemia in T2DM may have beneficial effects on MASLD, with a primary focus on glucose and lipid reduction (EASL-EASD-EASOEuropean Association for the Study of Diabetes EASDEuropean Association for the Study of Obesity EASO, 2016). However, a critical limitation of these drugs is their lack of precision therapy and etiological treatment, which ultimately results in suboptimal therapeutic outcomes. The objective of this study was to investigate the potential for identifying biomarkers and pathogenesis associated with T2DM combined with MASLD through the application of bioinformatics methods. This approach aims to facilitate the discovery of novel diagnostic and therapeutic targets for patients with T2DM combined with MASLD.

## 2 Materials and methods

### 2.1 Data collection

We obtained datasets for MASLD and T2DM from the GEO public database (http://www.ncbi.nlm.nih.gov/geo/). The MASLD dataset contains GSE33814 and GSE48452, where GSE33814 has 13 controls and 12 MASLD patients and GSE48452 contains 14 controls and 18 MASLD patients. The T2DM datasets GSE76895 containing 32 controls and 36 patients with T2DM; and GSE89120 containing 7 controls and 7 patients with T2DM.

### 2.2 Identification of differentially expressed genes (DEGs)

We merged the two datasets for each disease to increase the sample size. Data is normalized using R language to reduce heterogeneity. Then analyzed DEGs in the patient and control samples using the GEO2R software at an adjusted P < 0.05, with log_2_FC > 0.5 being upregulated and log_2_FC < −0.5 being downregulated for DEGs. Volcano maps and heat maps were created using the “ggplot2” and “heat map” packages.

### 2.3 Weighted gene coexpression network analysis (WGCNA)

WGCNA (Weighted Gene Co-expression Network Analysis) is a powerful algorithm designed to identify gene modules with significant biological relevance and explore their relationships with diseases. In this study, we used the “WGCNA” R software package to construct a co-expression network integrating clinical features of MASLD and T2DM. Following the scale-free network criterion, we used the “picksoft threshold” function in the “WGCNA” package to determine the optimal power value (ranging from 1 to 20). After selecting the best power value, we constructed the proximity matrix and adjusted the gene distribution to fit the scale-free network model based on connectivity. We then calculated the topological overlap matrix (TOM) and used it to re-cluster the genes. Finally, we computed the correlation coefficients and corresponding p-values between the different gene modules and clinical traits.

### 2.4 Enrichment analysis

Gene Ontology (GO) enrichment analysis of DEGs including molecular function (MF), cellular component (CC) and biological process (BP) using the DAVID online tool (https://david.ncifcrf.gov/home). Differential gene enrichment pathways were analyzed using the KOBAS website (http://bioinfo.org/kobas/).

### 2.5 Validation of the optimal hub gene

We constructed diagnostic models for MASLD and T2DM using least absolute shrinkage and selection operator (LASSO) regression (“glmnet” R package), respectively. Next, we took the intersection of the two outcomes predicted by the MASLD and T2DM models as a candidate diagnostic hub genes.

We evaluated the diagnostic value of the hub gene for both diseases by performing ROC analysis on the MASLD and T2DM datasets.The ROC analysis generated area under the curve (AUC) and 95% confidence intervals (CI), with an AUC value >0.7 considered to have strong diagnostic efficacy.

### 2.6 Animal model construction

Six-week-old male C57BL/6J mice were randomly divided into three groups (n = 5 or 6 mice/group): (1) standard diet-fed control group, SD group; (2) high-fat diet-fed up to 16 weeks to construct a MASLD model referring to zhu, et al. (Wang et al., 2024), HFD group; and (3) high-fat diet-fed and injected intraperitoneally with streptozotocin (STZ) (30 mg/kg) to construct a T2DM model (Li et al., 2024), HFD + STZ group. All mice housed in a specific pathogen-free facility with a 12-h light and 12-h dark cycle at room temperature. Body weight, and fasting blood glucose (FBG) levels were recorded biweekly. All experiments were conducted in accordance with the protocols approved by the Animal Research Committee of Tongji Medical College, Huazhong University of Science and Technology, Hubei Province, China (IACUC Number: 3994).

### 2.7 FBG, IPITT and IPGTT

The experimental steps were as previously described (Li et al., 2024), in brief, FBG levels in mice were determined using a blood glucose meter (LifeScan) following an overnight fast. The intraperitoneal insulin tolerance test (IPITT) was conducted by administering insulin (0.75 U/kg, intraperitoneally) after an overnight fast, and the intraperitoneal glucose tolerance test (IPGTT) was carried out by injecting glucose (2 g/kg, intraperitoneally) following another overnight fast. Plasma glucose concentrations were measured at various time intervals.

### 2.8 RT-qPCR

Total RNA was extracted from mouse pancreas and liver tissues by the TRIzol method. Then cDNA was synthesized using HiScript III RT SuperMix (Vazyme, China) under the following conditions: 15 min at 37°C and 5 s at 85°C. The gene sequence number (Rdh10 ID: 98711, Gapdh ID: 14433) was looked up on the NCBI website and primers were designed using Primer-BLAST. RT-qPCR was performed on a LightCycler 480 System with a LightCycler 480 SYBR Green I Master. The cDNA was then amplified using ChamQ SYBR qPCR Master Mix (Vazyme, China). Gapdh was used as an internal control in a volume of 10 μL. mRNA levels of the target genes were determined by RT-qPCR amplification and relative mRNA levels were calculated using the 2^−ΔΔCT^ method.

The primers (Tsingke Biotech, China) used for quantification of relative mRNA expression were as follows: *Gapdh-F:*AGGTCGGTGTGAACGGATTTG and *Gapdh-R:* TGT​AGA​CCA​TGT​AGT​TGA​GGT​CA, *Rdh10-F:* ATG​GTT​CGC​CAC​ATC​TAC​CG and *Rdh10-R*: CTC​CTC​ACC​TTT​TCC​AGC​TTG​C.

### 2.9 Western blotting

Proteins from liver and pancreas tissues were extracted using RIPA lysis buffer (NCE Biotech, China) supplemented with protease and phosphatase inhibitors. The protein samples were separated by SDS-PAGE and transferred to PVDF membranes (Millipore, United States). The membranes were incubated overnight at 4°C with primary antibodies: rabbit anti-RDH10 (1:1,000 dilution, Proteintech, 14644-1-AP, China) and mouse anti-β-actin (1:2,000 dilution, Proteintech, 66009-1-Ig, China). Afterward, they were incubated with secondary antibodies (1:3,000 dilution) for 1 h at room temperature. Protein bands were visualized and quantified using ECL reagent (NCE Biotech, China). The protein marker used was the 180 kDa Plus Prestained Protein Marker (Thermo, 26,616, United States).

### 2.10 Immunofluorescence staining

Paraffin-embedded liver and pancreas sections were deparaffinized with xylene and then progressively dehydrated in 100%, 95%, 85%, and 70% ethanol for 5 min each. The sections were incubated overnight at 4°C with primary antibodies: rabbit anti-RDH10 (1:1,000 dilution, Proteintech, 14644-1-AP, China), mouse anti-insulin (1:100 dilution, Proteintech, 66198-1-Ig), and rabbit anti-glucagon (1:100 dilution, Abcam, Ab92517). Following PBS washes, the sections were treated with secondary antibodies (FITC and CY3, Servicebio) for 1 h, and then stained with DAPI (Servicebio, Wuhan, China) at room temperature in the dark to label the nuclei. The stained sections were visualized using a fully automated section scanning system (VS120, Olympus, Japan).

### 2.11 Histological analysis and lipid content detection

Liver tissues were preserved in 4% paraformaldehyde, subsequently enclosed in paraffin blocks, and precisely sectioned into 5 μm slices. To assess hepatic histology, the liver sections underwent H&E staining. Additionally, the enzymatic standards from a diagnostic kit (Nanjing Jiancheng, China) were employed to determine the levels of triglycerides (TG) and total cholesterol (TC), low-density lipoprotein (LDL) and high-density lipoprotein (HDL) in the serum of different groups of mice.

### 2.12 Study population

The clinical samples involved in the experiment were sourced from our previous studies (Chen et al., 2023). We selected male subjects aged 45–65 years. Clinical sample selection criteria for the T2DM group: 1. Fasting plasma glucose (FPG) ≥ 7.0 mmol/L; 2. Random plasma glucose ≥11.1 mmol/L accompanied by classic diabetes symptoms (e.g., polyuria, polydipsia, weight loss, fatigue); 3. 2-h plasma glucose ≥11.1 mmol/L during an oral glucose tolerance test (OGTT); 4. HbA1c ≥ 6.5%; 5. A confirmatory test on a separate day is required to confirm the diagnosis. Clinical sample selection criteria for the MASLD group: 1. Presence of imaging or histologic evidence of hepatic steatosis; 2. Comorbidity with at least one metabolic disorder such as obesity, impaired glucose tolerance, hypertension, or dyslipidemia. Exclusion criteria were: 1. patients with severe renal insufficiency, severe hepatic insufficiency, decompensated heart failure, myocardial infarction, malignant neoplasm, severe infections, and other severe systemic diseases. 2. type 1 diabetes, type-specific diabetes, and insulin-dependent diabetes. 3. history of previous pancreatitis. 4. patients with Cushing’s Syndrome and patients with prior use of glucocorticoids. 5. Patients who have undergone gastrointestinal or other abdominal surgery within the past year. 6. Other liver diseases: exclude liver diseases due to other causes, including alcoholic liver disease, viral hepatitis, autoimmune liver disease, drug-induced liver injury, and hereditary liver disease. The study adhered to the guidelines of the Declaration of Helsinki and was approved by the Ethics Committee of Tongji Medical College, Huazhong University of Science and Technology (ChiCTR2000034751).

### 2.13 Enzyme-linked immunosorbent assay (ELISA)

Serum was obtained from human fasting peripheral blood and mouse blood collected from eye sockets by centrifugation (4°C, 1,500 g, 20 min) and stored at −80°C. The levels of RDH10 were determined using the Human and Mouse Retinol Dehydrogenase 10 (RD10) ELISA Kits (Jingkang, China) according to the manufacturer’s instructions. Absorbance at 450 nm was measured using an ELISA (PerkinElmer, Waltham, MA, United States).

### 2.14 Statistical analysis

Data were analyzed and processed using GraphPad Prism 9.5 software. Unpaired two-tailed t-test was used to assess the differences in numerical parameters between the two groups, and one-way or two-way ANOVA was used for multiple comparisons between groups. All data are expressed as mean ± standard deviation, and p < 0.05 was considered statistically significant.

## 3 Results

### 3.1 Identification of DEGs for MASLD and T2DM

**FIGURE 1:**
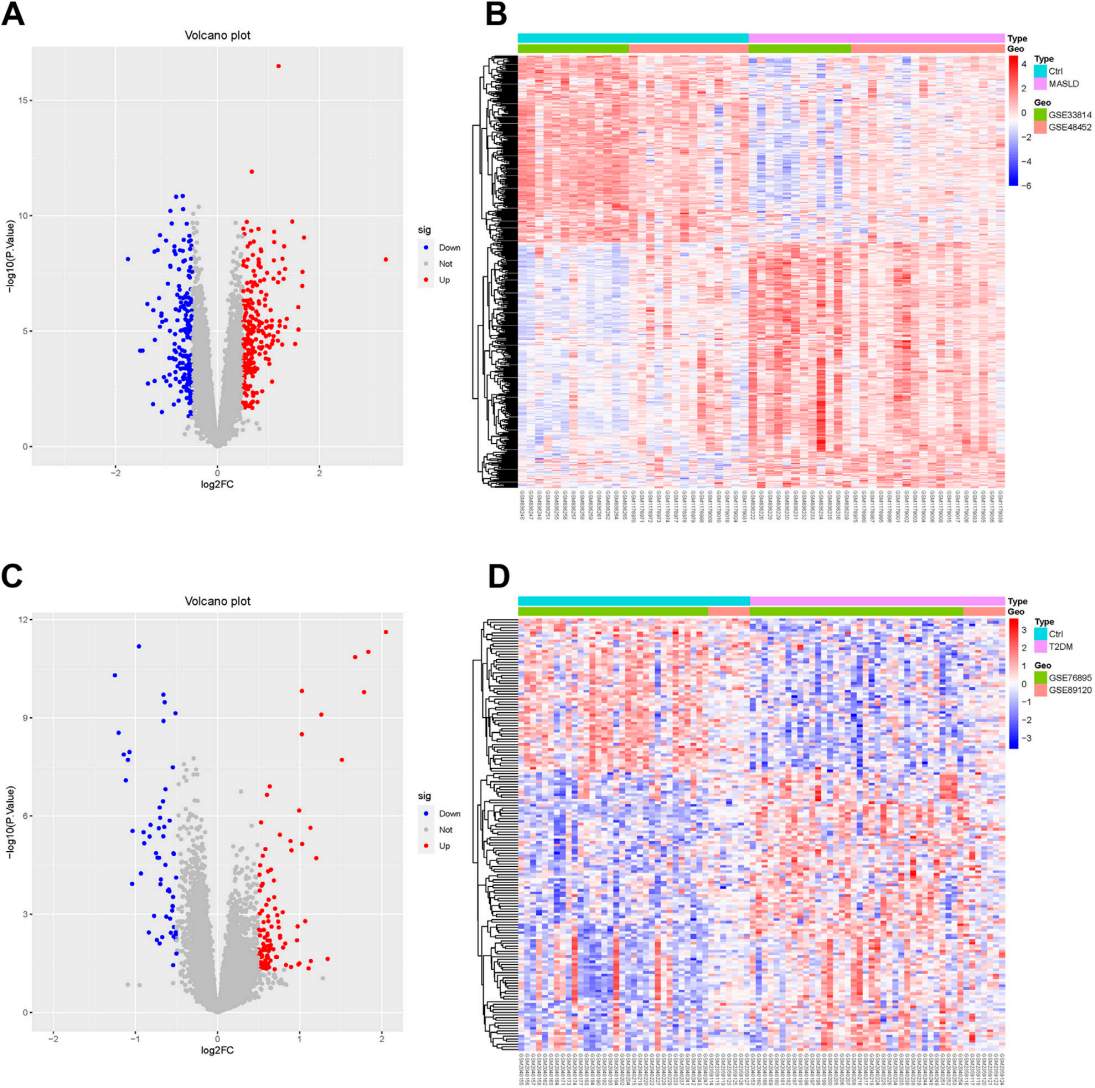
Identification of MASLD and T2DM differentially expressed genes (DEGs). **(A, C)** Volcano diagrams of MASLD and T2DM. **(B, D)** Heatmaps showing expression patterns of DEGs in MASLD and T2DM, normalized and clustered to reveal disease-specific transcriptional profiles.

We downloaded the MASLD dataset (GSE33814 and GSE48452) from the Gene Expression Omnibus (GEO) database and performed data cleaning and analysis to identify 439 DEGs, including 249 upregulated genes, 190 downregulated genes. We plotted these results as volcano and heat maps (Figures 1A, B).

Performing the same operation on the T2DM dataset (GSE76895 and GSE89120), we identified 163 DEGs, of which 105 were upregulated and 58 downregulated. The results are shown in the volcano and thermograms of Figures 1C, D.

### 3.2 WGCNA

**FIGURE 2:**
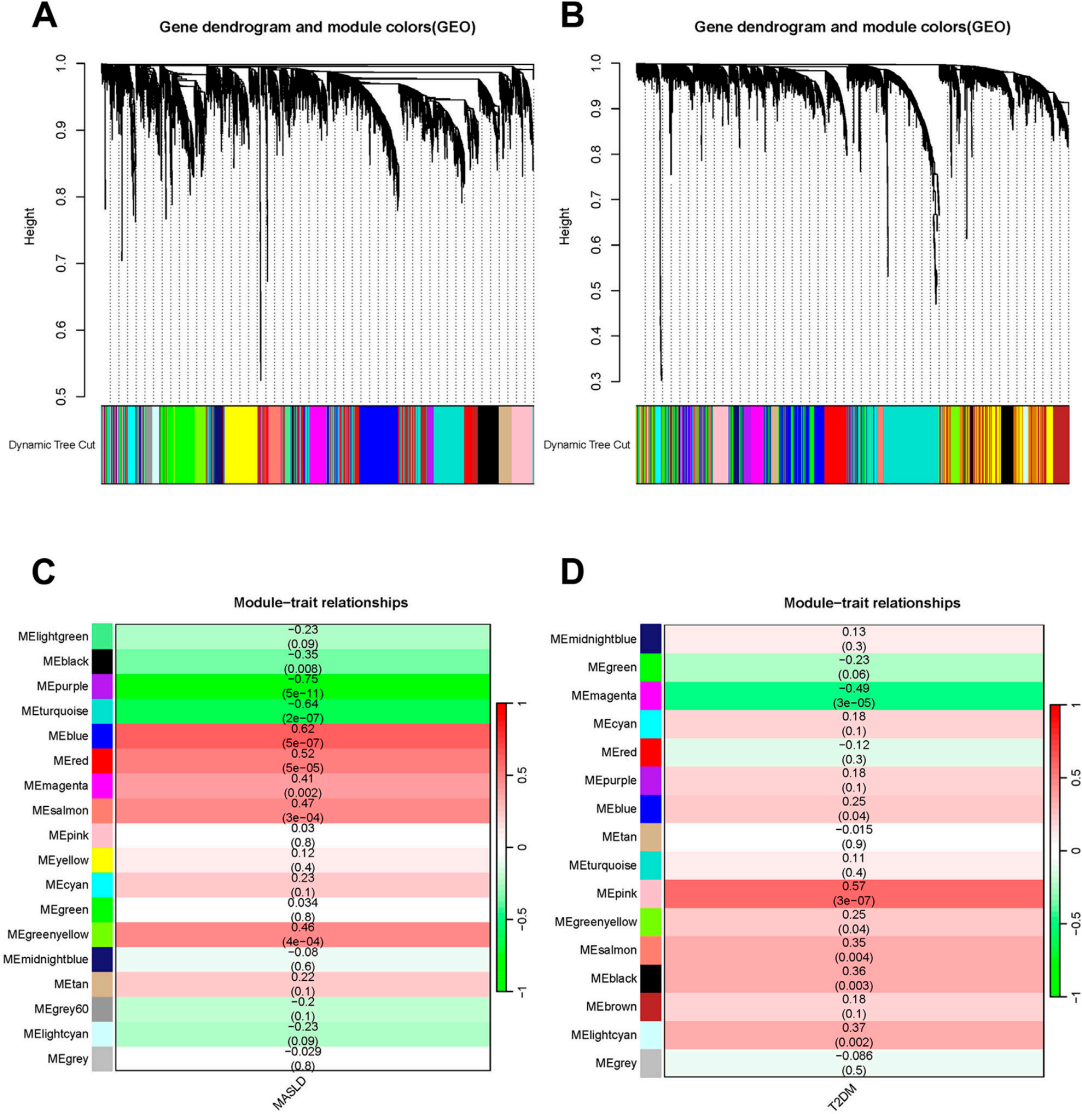
Weighted analysis of gene co-expression networks. **(A)** The MASLD differentially expressed genes (DEGs) cluster dynamics dendrogram. **(B)** MASLD module-trait correlations. Matching correlations and p-values are provided in each cell. **(C)** The DEGs of T2DM are shown in a cluster dynamic dendrogram. **(D)** Module-trait correlations for T2DM. Matching correlations and p-values are provided in each cell.

We used WGCNA to detect clusters of co-expressed genes differentially expressed between MASLD and T2DM and assessed the relevance of combinatorial modules to disease phenotypes. A total of 18 modules of MASLD were identified through hierarchical clustering, in which the module positively correlated with the incidence of MASLD was blue (p < 0.001), and the module negatively correlated was purple (p < 0.001) (Figures 2A, C). The T2DM model centrally identified 16 modules, of which the pink module (p < 0.001) was highly positively correlated and the magenta module (p < 0.001) was negatively correlated with the incidence of T2DM (Figures 2B, D).

### 3.3 Functional enrichment analysis of co-DEGs of MASLD and T2DM

**FIGURE 3:**
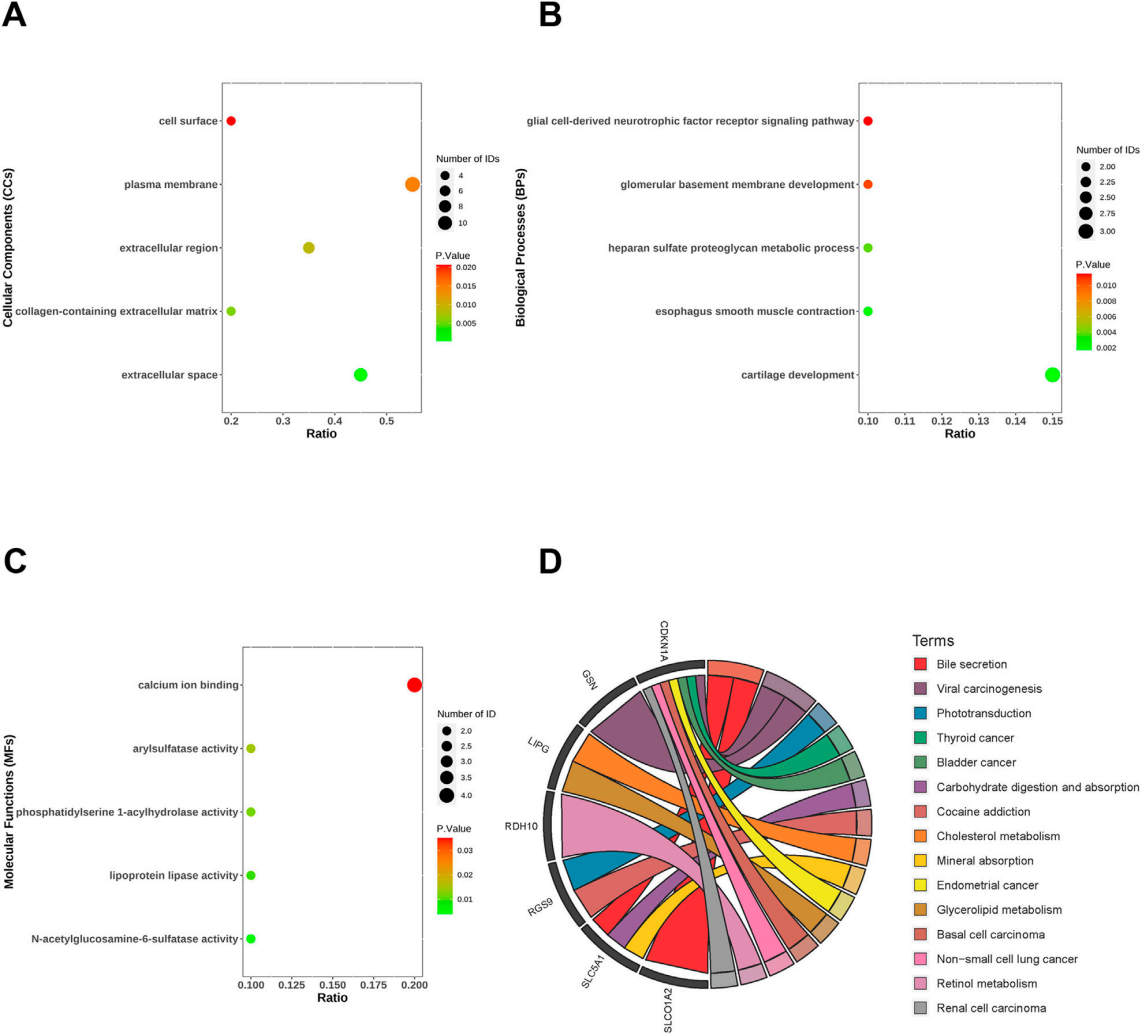
Functional enrichment analysis of co-differentially expressed genes (co-DEGs). Cellular components **(A)**, biological processes **(B)**, molecular functions **(C)** and KEGG **(D)** of co-DEGs.

The most highly positively and negatively correlated module for MASLD and T2DM has 20 genes overlapping. These genes may regulate both MASLD and T2DM development, we analyzed the biological processes (BPs), cellular components (CCs), and molecular functions (MFs) and enrichment pathways of these genes. In the CCs, co-DEGs were mainly enriched in extracellular space, collagen-containing extracellular matrix, extracellular region, plasma membrane, cell surface (Figure 3A). The BPs were mainly focused on cartilage development, esophagus smooth muscle contraction, heparan sulfate proteoglycan metabolic process, glomerular basement membrane development and glial cell-derived neurotrophic factor receptor signaling pathway (Figure 3B). As for the MFs group, DEGs were mainly enriched in N-acetylglucosamine-6-sulfatase activity, lipoprotein lipase activity, phosphatidylserine 1-acylhydrolase activity, arylsulfatase activity, calcium ion binding (Figure 3C). KEGG pathway analysis revealed that the DEGs were mainly focused on the bile secretion, phototransduction, cancer, carbohydrate digestion and absorption, cholesterol/glycerol metabolism, and retinol metabolism (Figure 3D).

### 3.4 Identification of candidate diagnostic biomarkers

**FIGURE 4:**
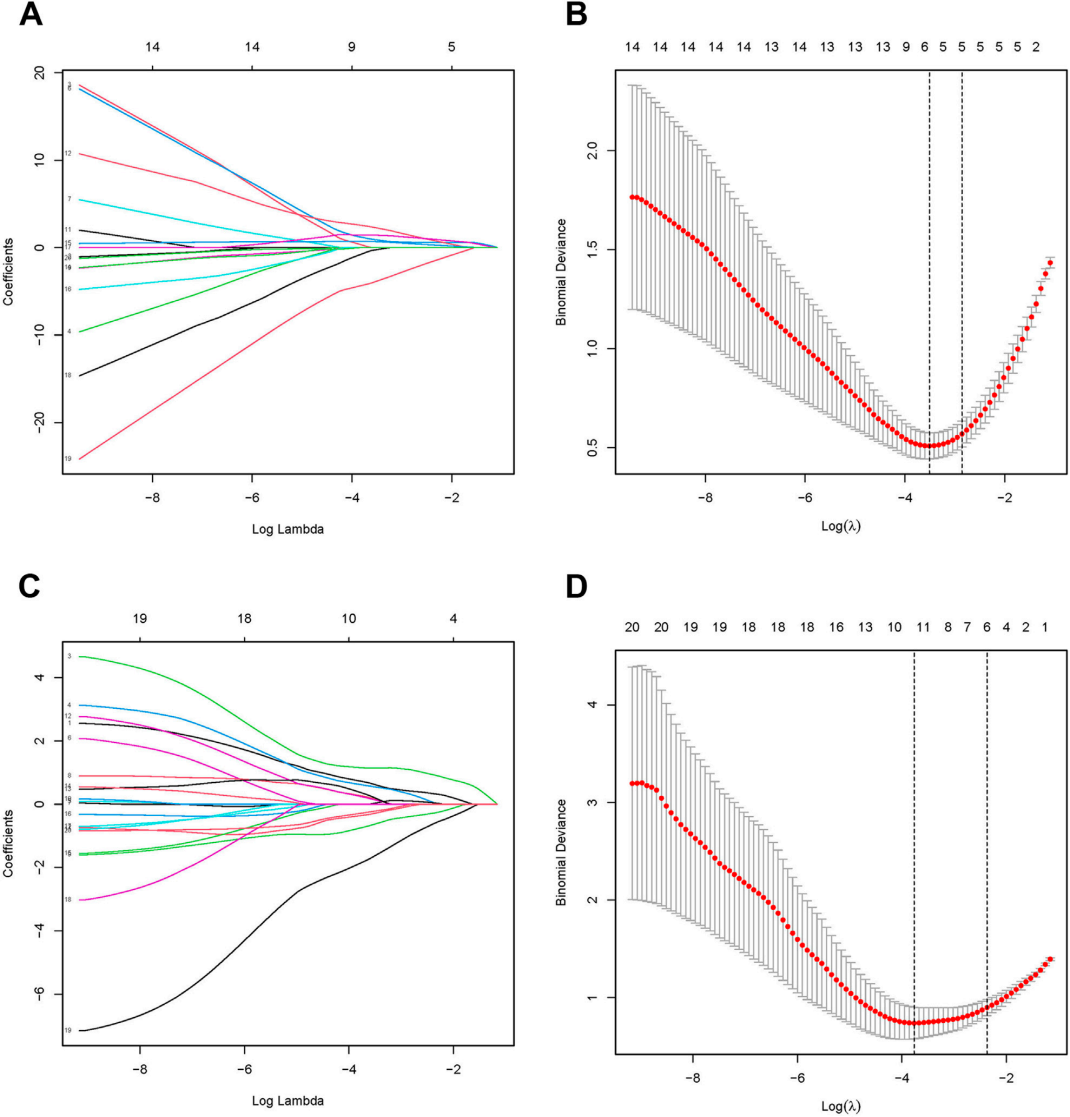
Identification of candidate diagnostic biomarkers. The Least Absolute Shrinkage and Selection Operator (LASSO) algorithm screens MASLD **(A, B)** and T2DM **(C, D)** for optimal diagnostic genes. LASSO identifies genes with the strongest predictive power, highlighting potential biomarkers for accurate diagnosis. In the LASSO, λ (lambda) represents the regularization parameter that controls the strength of penalty applied to the model during the optimization process, effectively performing feature selection.

We used the LASSO algorithm to identify the predictive value of candidate genes. We extracted the expression data of 20 genes from the MASLD gene expression profile using the LASSO algorithm, and the optimal lambda value was lambda.min = 0.0299 (Figures 4A, B).The LASSO regression results revealed that there are 6 genes as feature genes in MASLD. We used the same method to obtain 10 diagnostic genes for T2DM(lambda.min = 0.0232, Figures 4C, D). We intersected the diagnostic genes for MASLD and T2DM to obtain 2 genes, namely, *SATB2, RDH10*.

### 3.5 Validating the diagnostic value of differential genes

**FIGURE 5:**
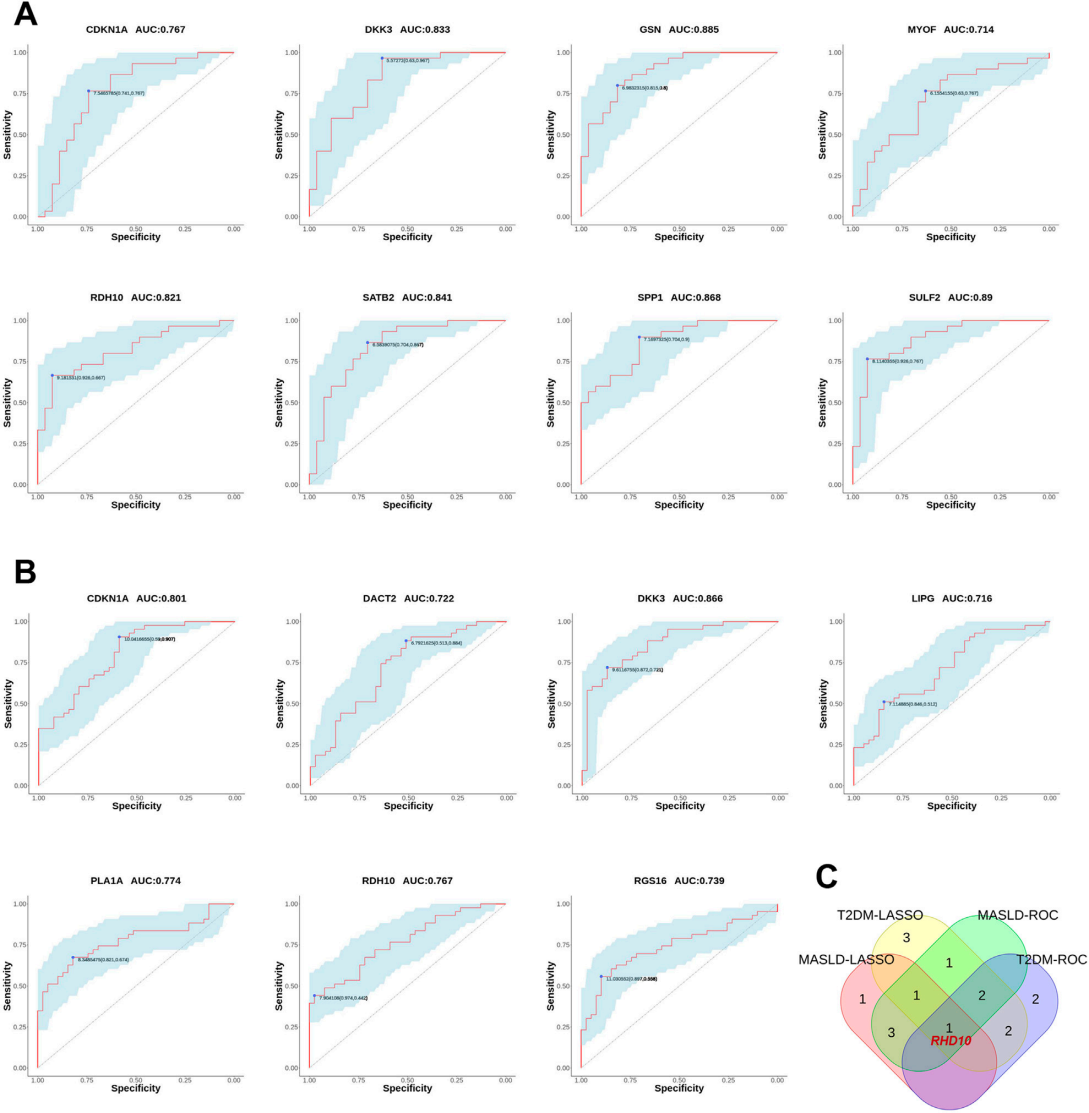
Validating the diagnostic value of differential genes. **(A)** The receiver operating characteristic (ROC) curves for MASLD candidate genes. **(B)** ROC curves for T2DM candidate genes. **(C)** Venn diagram showing the co-differentially expressed genes (co-DEGs) of MASLD and T2DM were passed through the least absolute shrinkage and selection operator (LASSO) algorithm and ROC curves intersected to obtain hub genes. In ROC analysis, the area under the curve (AUC) represents the predictive ability, and a higher AUC value indicates a better predictive value. In this study, AUC = 0.7 was chosen as the screening threshold.

We also plotted ROC curves for the pivotal genes and calculated the AUC to assess the diagnostic value of the DEGs. The diagnostic values of hub genes in MASLD were as follows: *SULF2* (AUC, 0.89), *GSN* (AUC, 0.885), *SPP1* (AUC, 0.868), *SATB2* (AUC, 0.841), *DKK3* (AUC, 0.833), *RDH10* (AUC, 0.821), *CDKN1A* (AUC, 0.767), *MYOF* (AUC, 0.714) (Figure 5A). Similarly, we verified the diagnostic value of these genes for T2DM as follows: *DKK3* (AUC, 0.866), *CDKN1A* (AUC, 0.801), *PLA1A* (AUC, 0.774), *RDH10* (AUC, 0.767), *RGS16* (AUC, 0.739), *DACT2* (AUC, 0.722), *LIPG* (AUC, 0.716) (Figure 5B). The AUCs of the DEGs in both MASLD and T2DM were screened by ROC curves to be greater than 0.7 names as *CDKN1A, DKK3, RDH10*. Intersecting the DEGs obtained by the two algorithms, we finally identified *RDH10* as the best diagnostic gene for MASLD and T2DM (Figure 5C).

### 3.6 Establishment of MASLD and T2DM mouse models

Based on bioinformatics analysis, we found that *RDH10* expression was significantly downregulated in the pancreas of T2DM patients and the liver of MASLD patients. To further validate the changes of *Rdh10* in MASLD and T2DM, we constructed a MASLD mouse model using high-fat feeding (HFD) for 4 months. In addition, we induced mice with streptozotocin (STZ) to assess their diabetic status.

**FIGURE 6:**
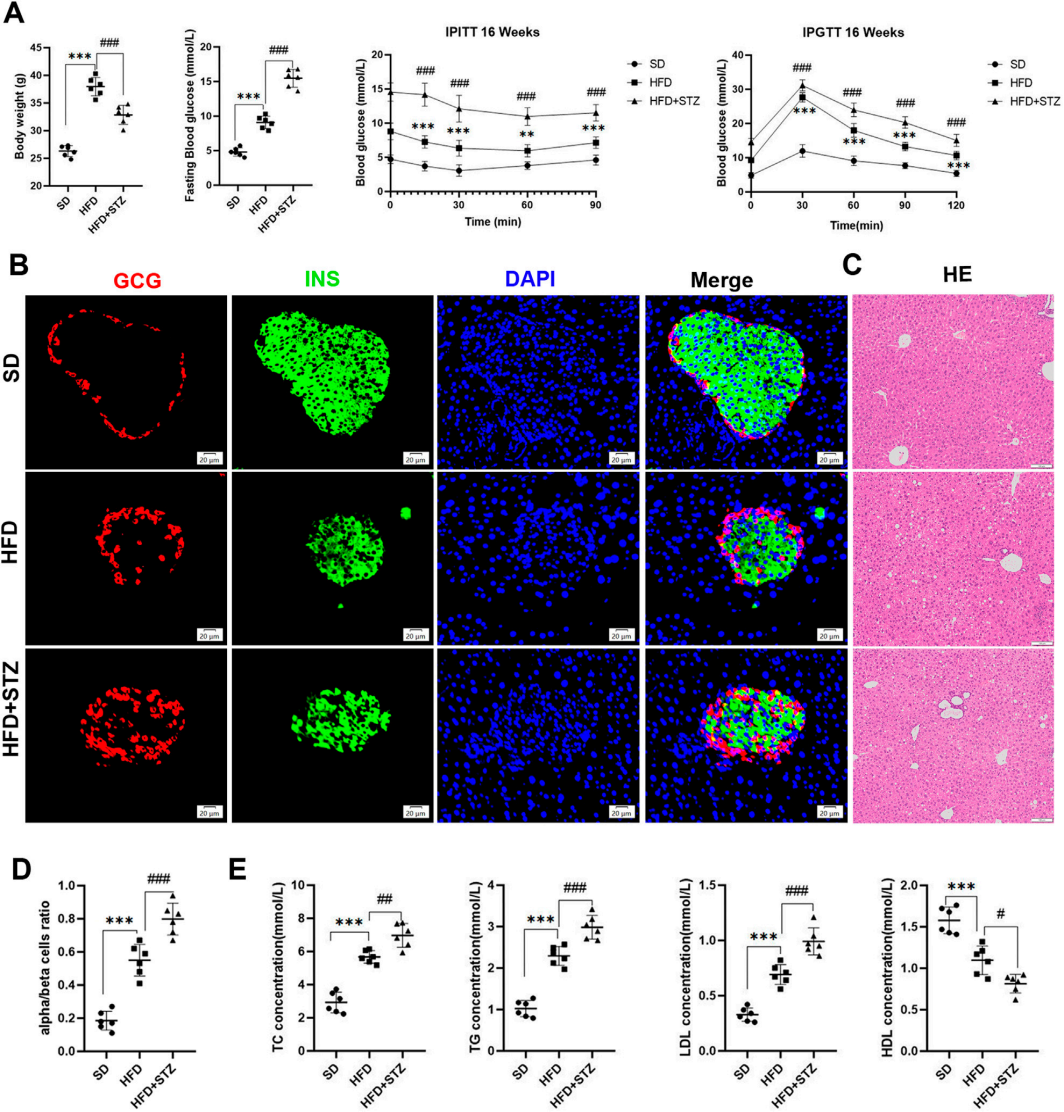
Establishment of MASLD and T2DM mouse models. **(A)** Body weight, fasting blood glucose, intraperitoneal insulin tolerance test (IPITT) and intraperitoneal glucose tolerance test (IPGTT) in different three groups of mice. **(B)** Double immunofluorescence of pancreatic glucagon (red) and insulin (green) in SD, HFD and STZ group, bar = 20 μm. **(C)** HE staining of the livers of three groups of mice, bar = 100 μm. **(D)** Quantification of pancreatic α/β cell ratio.**(E)** Serum levels of TC, TG, LDL, HDL in three groups of mice. **p < 0.01, ***p < 0.001 in SD vs. HFD; #p < 0.05, ##p < 0.01, ###p < 0.001 in HFD vs. STZ.

We observed a significant increase in body weight and blood glucose levels in HFD group with impaired glucose tolerance and insulin resistance compared to SD group (Figures 6A, B). As expected, histologic examination of liver tissues in the HFD group revealed marked swelling of hepatocytes and prominent microcystic steatosis by HE staining. Consistently, biochemical analyses showed that serum TG and hepatic TC levels were significantly elevated in the HFD group compared with the SD group. In addition, plasma lipid analyses showed that LDL levels were substantially elevated in the HFD group, whereas HDL levels were significantly reduced (Figures 6C, E).

STZ group further exacerbated HFD-induced elevated fasting glucose, abnormal glucose tolerance, and insulin resistance, with loss of pancreatic islet β-cells, increase in α-cells, and α/β-cell ratio imbalanced (Figures 6A, B, D). In addition, we observed increased hepatic steatosis, significantly increased serum TC,TG, and plasma LDL, and decreased HDL in STZ group compared to HFD group (Figures 6C, E).

### 3.7 RDH10 is downregulated in the pancreas and liver of MASLD and T2DM mouse models

**FIGURE 7:**
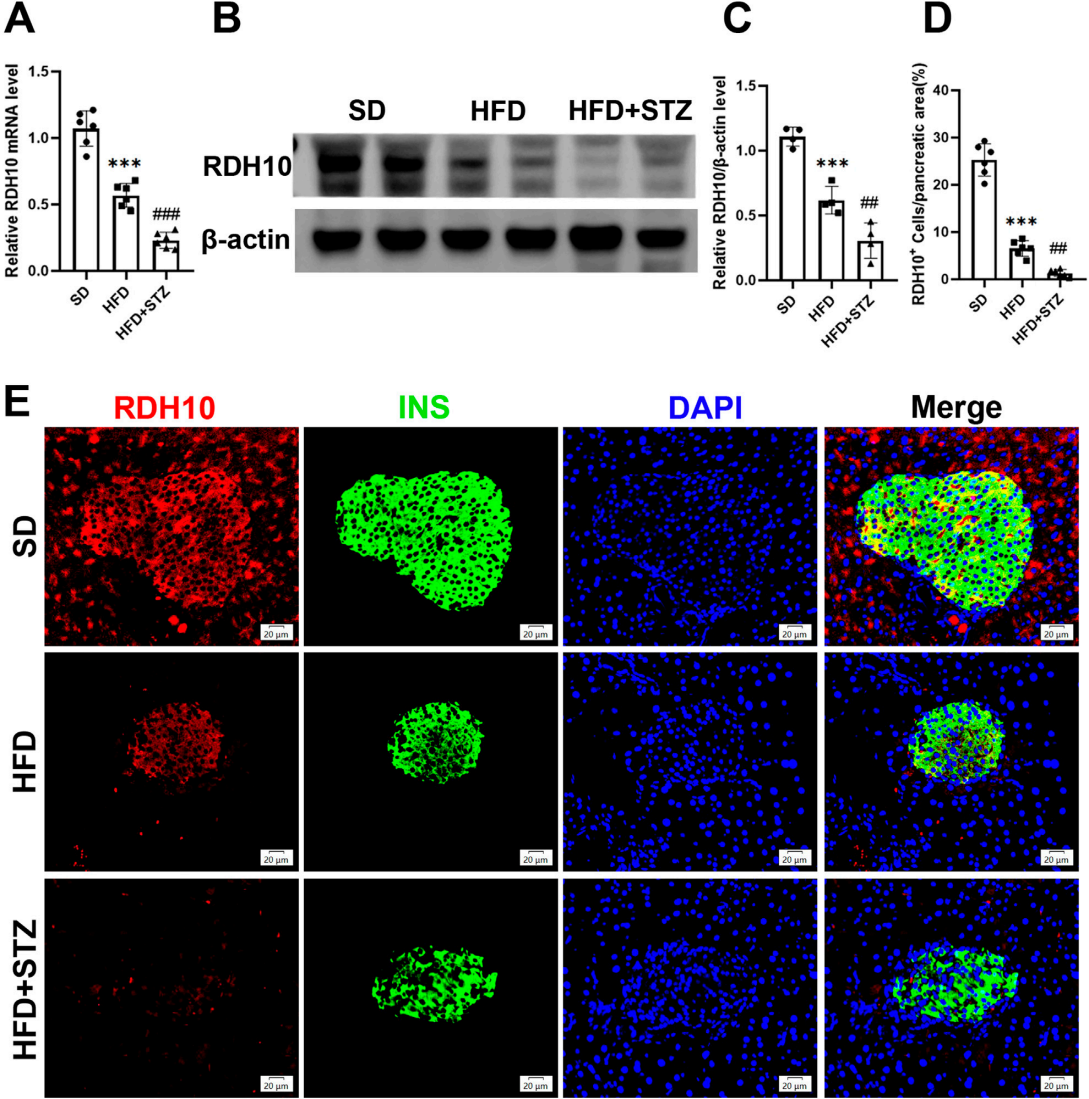
RDH10 is downregulated in the pancreas of MASLD and T2DM mouse models. **(A)** Quantitative analysis of RT-qPCR results of pancreatic *Rdh10* in different groups of mice. **(B, C)** RDH10 protein expression in the pancreas and quantitative analysis. **(D, E)** Representative pancreatic RDH10 (red) and insulin (green) double fluorescence staining images and quantitative analysis, bar = 20 μm ***p < 0.001 in SD vs. HFD; ##p < 0.01, ###p < 0.001 in HFD vs. STZ.

To validate the changes of RDH10 in MASLD and T2DM, we evaluated its expression levels in pancreas and liver. PCR results showed that *Rdh10* mRNA in the mouse pancreas was significantly reduced in both HFD and STZ groups, with a more remarkable decrease in STZ group (Figure 7A). Protein expression results are consistent with transcript levels, Western blot showing a significant decrease in RDH10 protein expression in the pancreas of both the HFD and STZ groups compared to the SD group (Figures 7B, C). Immunofluorescence of pancreatic islets showed that RDH10 was expressed in both pancreatic tissues and islets under physiological conditions, and with altered metabolic conditions, the expression of RDH10 was reduced firstly in the pancreas. Notably, RDH10 expression in STZ mice was not only decreased in the pancreas, but also significantly decreased in the pancreatic islets (Figures 7D, E).

**FIGURE 8:**
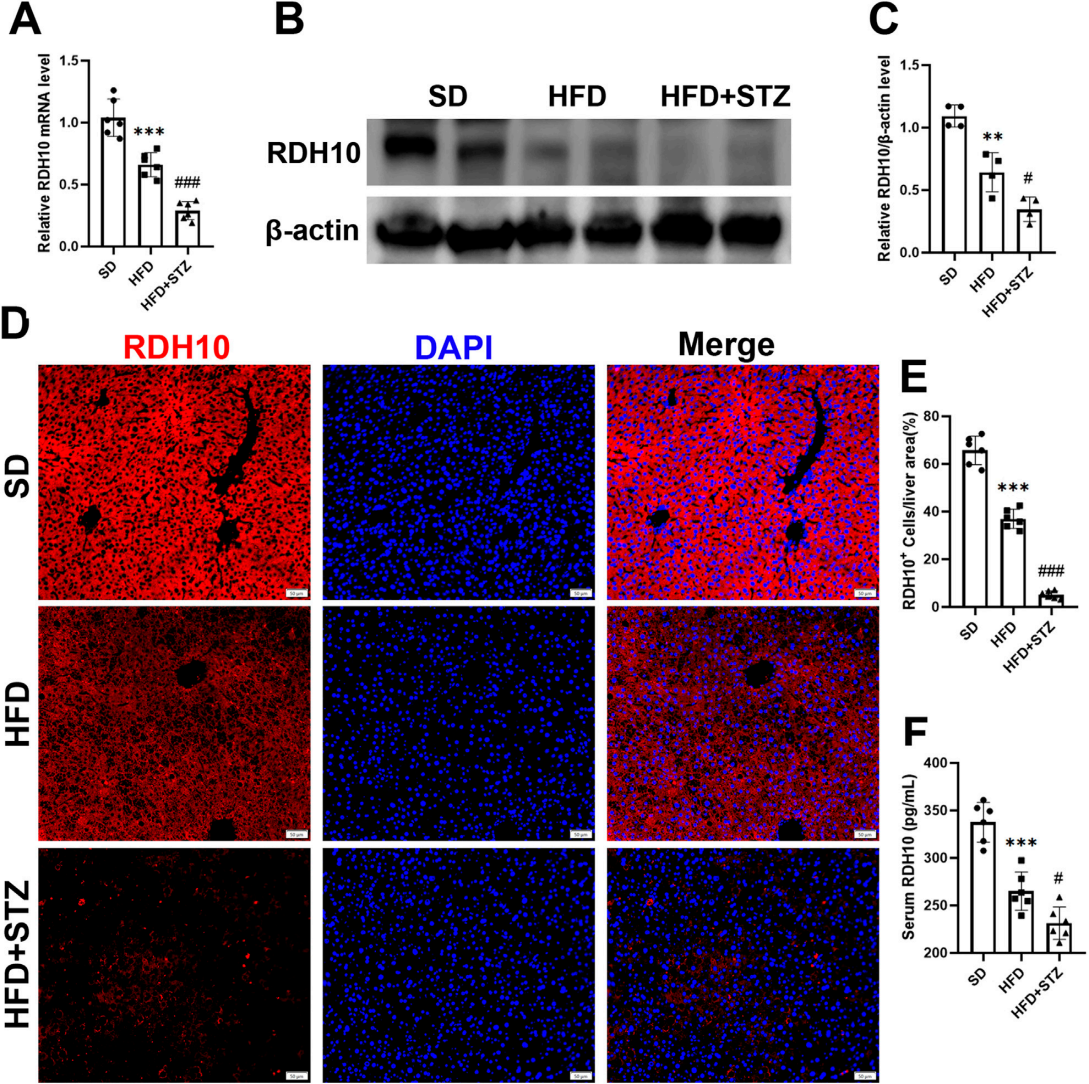
RDH10 is downregulated in the livers of MASLD and T2DM mouse models. **(A)** Quantitative analysis of RT-qPCR results of *Rdh10* in the livers of different groups of mice. **(B, C)** RDH10 protein expression in the livers and quantitative analysis. **(D, E)** Representative immunofluorescence images of RDH10 (red) in the livers of three groups of mice and quantitative analysis, bar = 50 μm. **(F)** Comparison of serum RDH10 expression in mice. **p < 0.01, ***p < 0.001 in SD vs. HFD; #p < 0.05, ###p < 0.001 in HFD vs. STZ.

Similarly, Transcript level validation showed that hepatic *Rdh10* mRNA was significantly reduced in the HFD and STZ groups, and further exacerbated in the STZ group (Figure 8A). RDH10 expression was reduced in the livers of the HFD and STZ groups, with a more significant reduction in the STZ group (Figures 8B, C). Immunofluorescence results of liver showed that RDH10 was normally expressed in most hepatocytes and gradually decreased with increasing metabolic burden (Figures 8D, E). Especially in the STZ group, RDH10 expression in the liver decreased significantly, compared with the SD group (Figures 8D, E).

In addition, we used ELISA to assess serum RDH10 levels in different groups of mice, and RDH10 was significantly lower in the HFD and STZ groups than in the SD group (Figure 8F).

### 3.8 Validation of RDH10 in clinical samples

**FIGURE 9:**
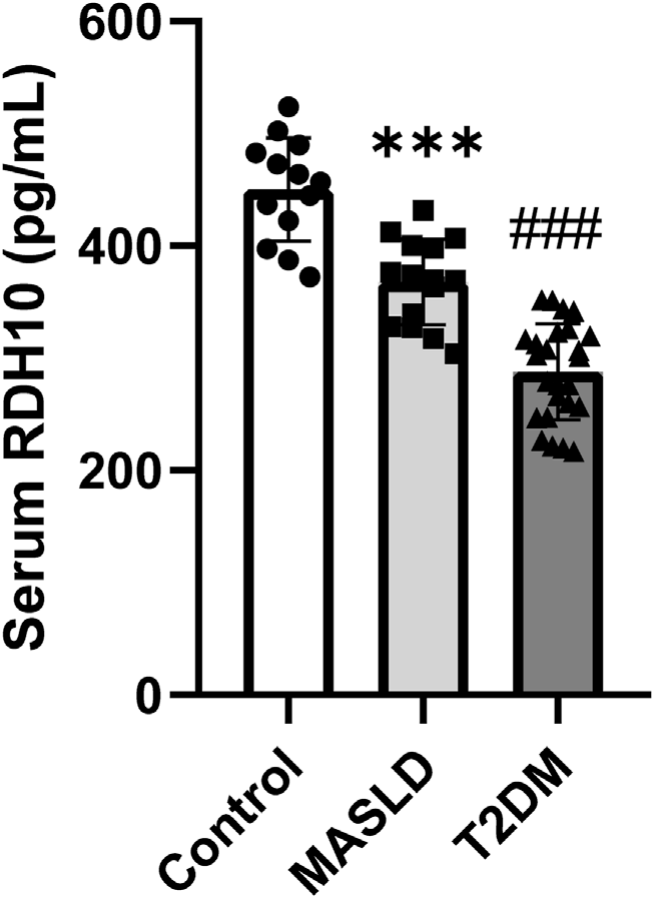
Comparison of serum RDH10 in three groups of volunteers. ***p < 0.001 in Control vs. MASLD; ###p < 0.001 in MASLD vs. T2DM.

To validate the clinical potential of RDH10, we included 13 controls, 15 patients with MASLD and 25 patients with T2DM to assess serum RDH10 expression. There was a statistically significant reduction in serum RDH10 in the MASLD group compared to the control group (Figure 9). Similarly, there was a statistically significant reduction in serum RDH10 in the T2DM group compared to the MASLD group (Figure 9). These findings suggest that RDH10 expression changes in the early stages of metabolic disorders and decreases with increasing metabolic stress.

## 4 Discussion

MASLD is significantly correlated with an increased risk of cardiometabolic disorders, particularly T2DM (European Association for the Study of the Liver EASLEuropean Association for the Study of Diabetes EASDEuropean Association for the Study of Obesity EASO, 2024). A recently published study has identified MASLD as a significant risk factor for new-onset T2DM and has demonstrated that it exhibits a higher predictive power for T2DM onset compared to conventional NAFLD (Sakai et al., 2024). Therefore, early identification of MASLD in the normal population is essential for the prevention of T2DM. Here, We screened differential genes associated with MASLD and T2DM by bioinformatics and constructed co-expression networks based on WGCNA to identify co-DEGs strongly positively and negatively associated with the two diseases. Using the LASSO algorithm and ROC curves to predict the diagnostic value of these co-DEGs, we finally identified *RDH10* as the most promising gene.

RDH10 is the major retinol dehydrogenase involved in retinoic acid biosynthesis and regulates glycolipid metabolism by maintaining vitamin A-like homeostasis (Sandell et al., 2007; Strate et al., 2009). As a biologically active derivative of vitamin A, retinoic acid is a vital component of the embryonic development process (Al Tanoury et al., 2013). In this study, by analyzing the co-DEGs of MASLD and T2DM, we found that these genes were significantly enriched in the retinol metabolism pathway, a result consistent with the critical role of RDH10 in retinol metabolism. It has been demonstrated that embryonic fibroblasts with *Rdh10* knocked down exhibited a reduction in retinoic acid biosynthesis and impaired retinoic acid signaling leading to developmental abnormalities (Rhinn et al., 2011). This not only highlights the central role of *Rdh10* in retinoic acid synthesis and signaling, but also suggests its importance during embryonic development. In HepG2 cells, the overexpression of RDH10 resulted in significant antiproliferative effects that were comparable to those exhibited by retinoids (Rossi et al., 2007). This finding further supports the potential role of RDH10 in the regulation of cell proliferation and metabolism.

Metabolic remodeling linked to RDH10 expression changes influences systemic metabolic states (Klyuyeva et al., 2021; Zhang et al., 2015). Cardiomyocyte-specific RDH10 knockout (RDH10-cKO) mice exhibit halved retinoic acid levels, heart failure, and severe cardiac remodeling, which AAV9-RDH10 injection mitigates (Wu et al., 2023). In addition, decreased expression of RDH10 increased fat deposition, uptake of FFAs, and TG levels in the myocardium of *db*/*db* mice, leading to cardiac lipotoxicity, a process that was antagonized by overexpression of RDH10 (Wu et al., 2023). Elimination of one *Rdh10* copy (*Rdh10* ^+/−^) increased adiposity, hepatic steatosis, glucose intolerance and insulin resistance in male mice fed HFD (Yang et al., 2018). These results suggest an important role for RDH10 in regulating glycolipid metabolism and preventing metabolism-related diseases. Consistent with these findings, our study showed that RDH10 expression was significantly downregulated in both liver and pancreas of MASLD and T2DM mouse models, with lower expression levels in the diabetic group. By immunofluorescence, we found that RDH10 was normally expressed in pancreatic tissues and pancreatic islets, and the expression gradually decreased with the aggravation of glycolipid disorders, and the expression of RDH10 was significantly reduced in the pancreas of T2DM mice. The downregulation of RDH10 in the T2DM pancreas may be related to the reduction of β-cells, and when pancreatic β-cells are exposed to hyperglycemia or high-fat environments, the increase of oxidative stress and inflammatory factors may inhibit the expression of RDH10.The downregulation of RDH10 further leads to the reduction of retinoic acid production, which affects the normal function of pancreatic β-cells and the secretion of insulin, further aggravating the deterioration of islet function, creating a vicious circle (Arregi et al., 2016). Similarly, in the physiological state, RDH10 was expressed in most hepatocytes of the liver, and with increasing metabolic disorders, RDH10 expression decreased significantly, with the most significant decrease in T2DM mice. Furthermore, serum levels of RDH10 were significantly lower in MASLD and T2DM model mice and humans than in controls.

The main limitation of this study is the small sample size, which may limit the statistical robustness and generalizability of the results. We will intend to further expand the sample size for testing. Differences in sequencing platforms or data processing methods may lead to systematic bias, although we used standardized methods to reduce heterogeneity. In addition, the role of RDH10 knockdown or overexpression in glycolipid disorder diseases and its molecular mechanisms need to be further explored. The protective role of RDH10 in ameliorating MASLD and T2DM by modulating retinol metabolism as well as other downstream molecules and thus needs to be validated by future experiments.

In conclusion, these results suggest an important role for RDH10 in glycolipid metabolism as a novel biomarker for MASLD and T2DM.

## Data Availability

Publicly available datasets were analyzed in this study. This data can be found here: http://www.ncbi.nlm.nih.gov/, accession numbers: GSE33814, GSE48452, GSE76895 and GSE89120.
