# Supplementary figures and images for: Identification of retinol dehydrogenase 10 as a shared biomarker for metabolic dysfunction-associated steatotic liver disease and type 2 diabetes mellitus
Source: Front Pharmacol. 2025 Jan 24;16:1521416. doi: 10.3389/fphar.2025.1521416 (PMC11802817; doi:10.3389/fphar.2025.1521416)

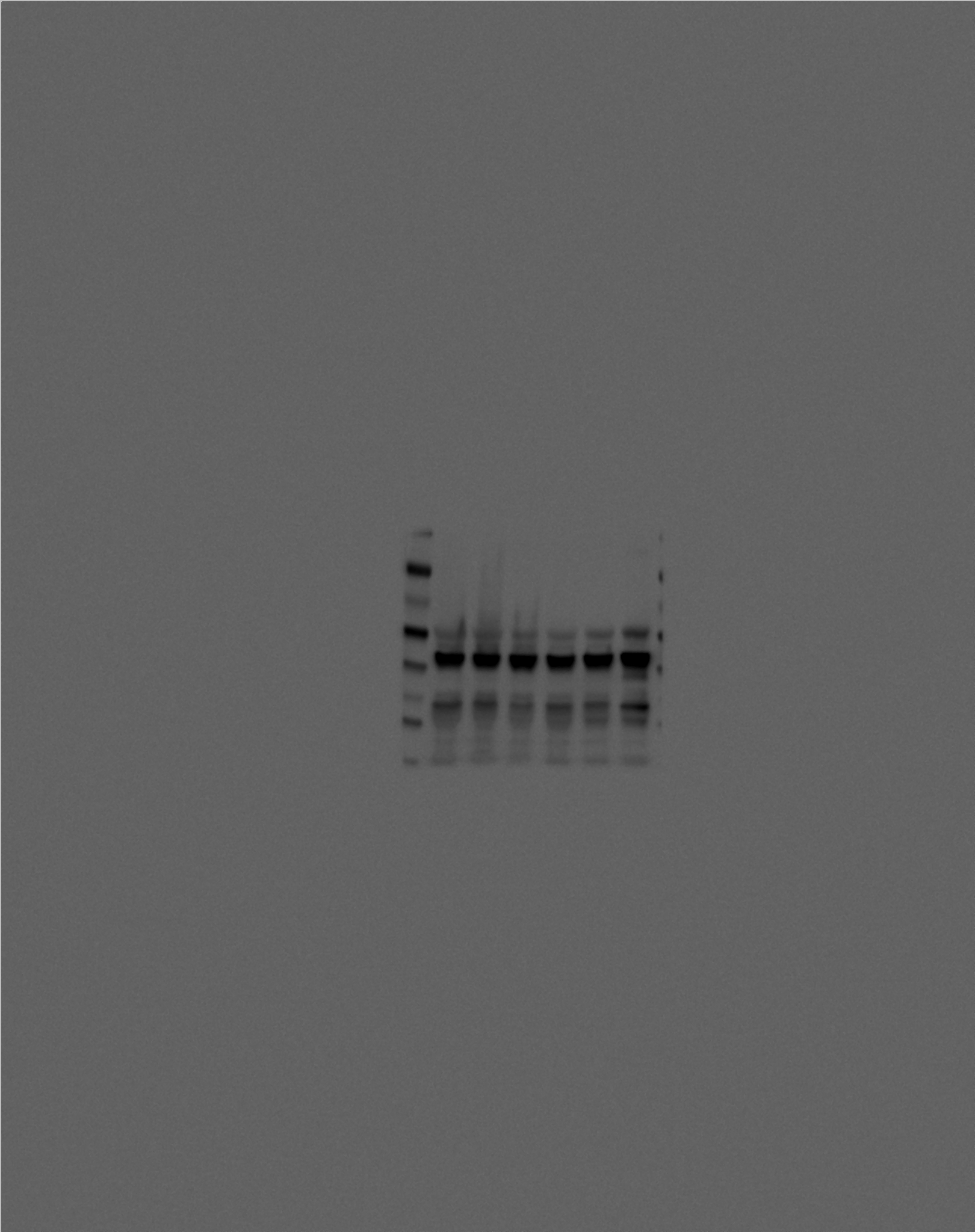

Supplement: Supplementary file 1 [file DataSheet1.zip › data/islet/1actin.jpg]

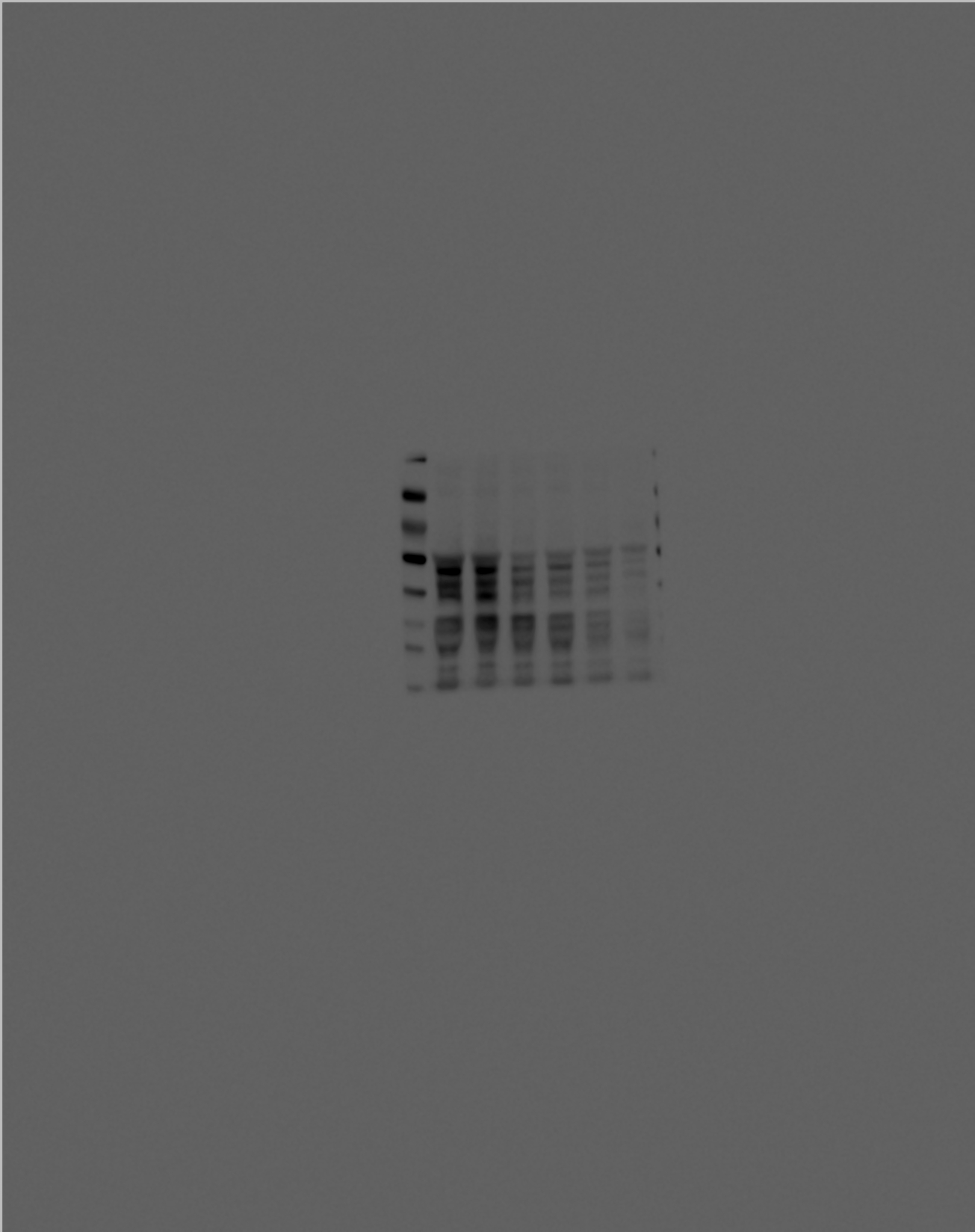

Supplement: Supplementary file 1 [file DataSheet1.zip › data/islet/1rdh10.jpg]

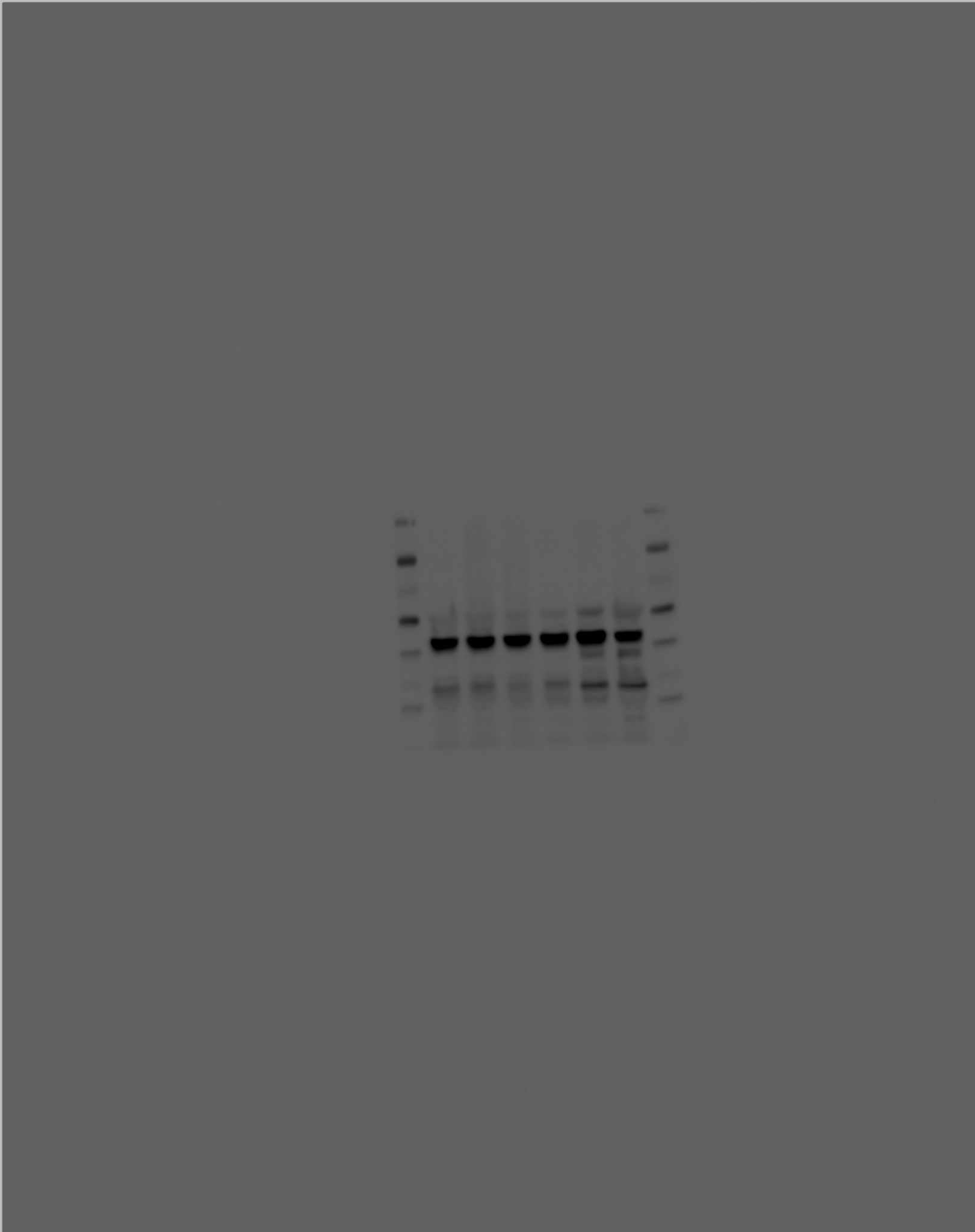

Supplement: Supplementary file 1 [file DataSheet1.zip › data/islet/2actin.jpg]

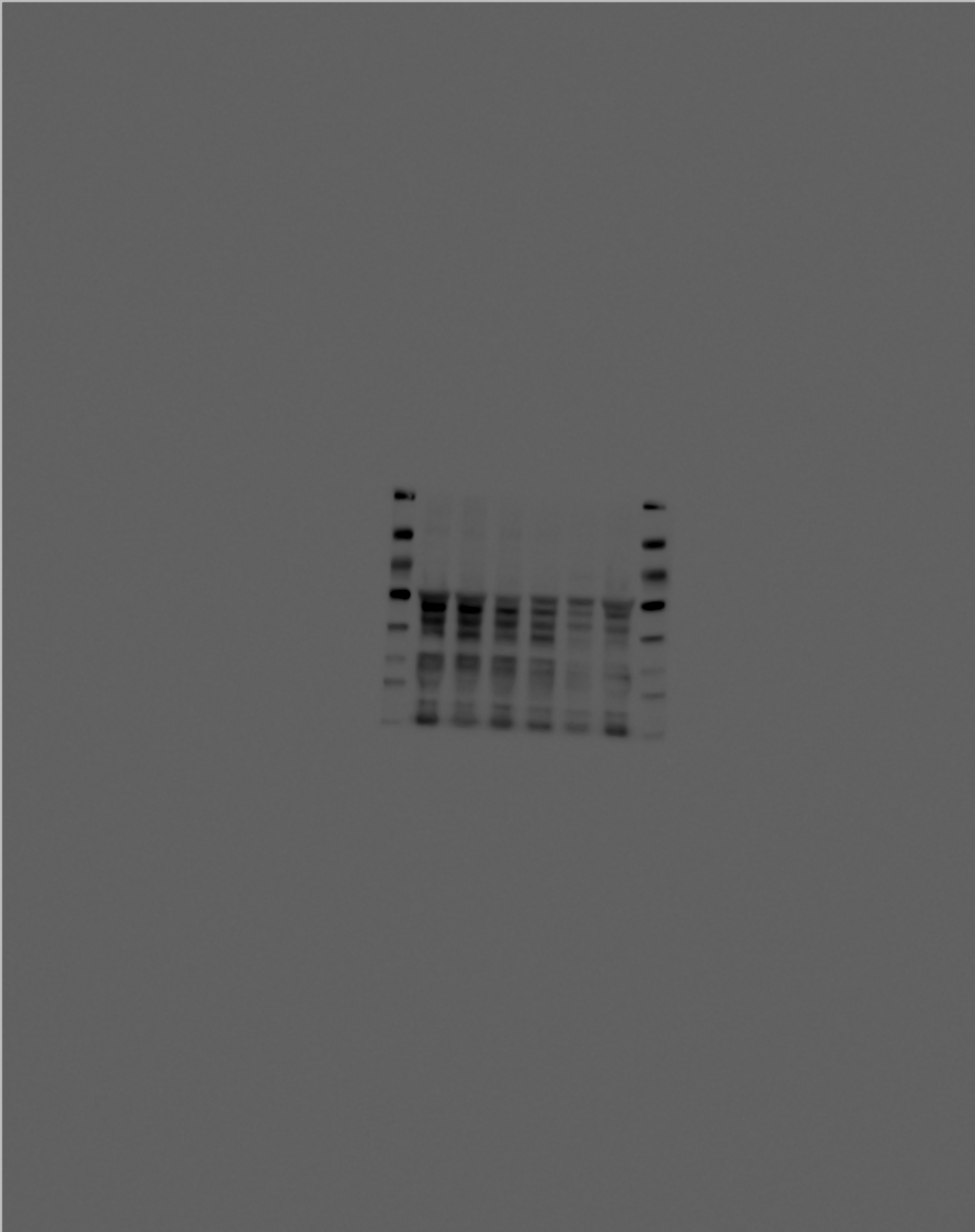

Supplement: Supplementary file 1 [file DataSheet1.zip › data/islet/2rdh10.jpg]

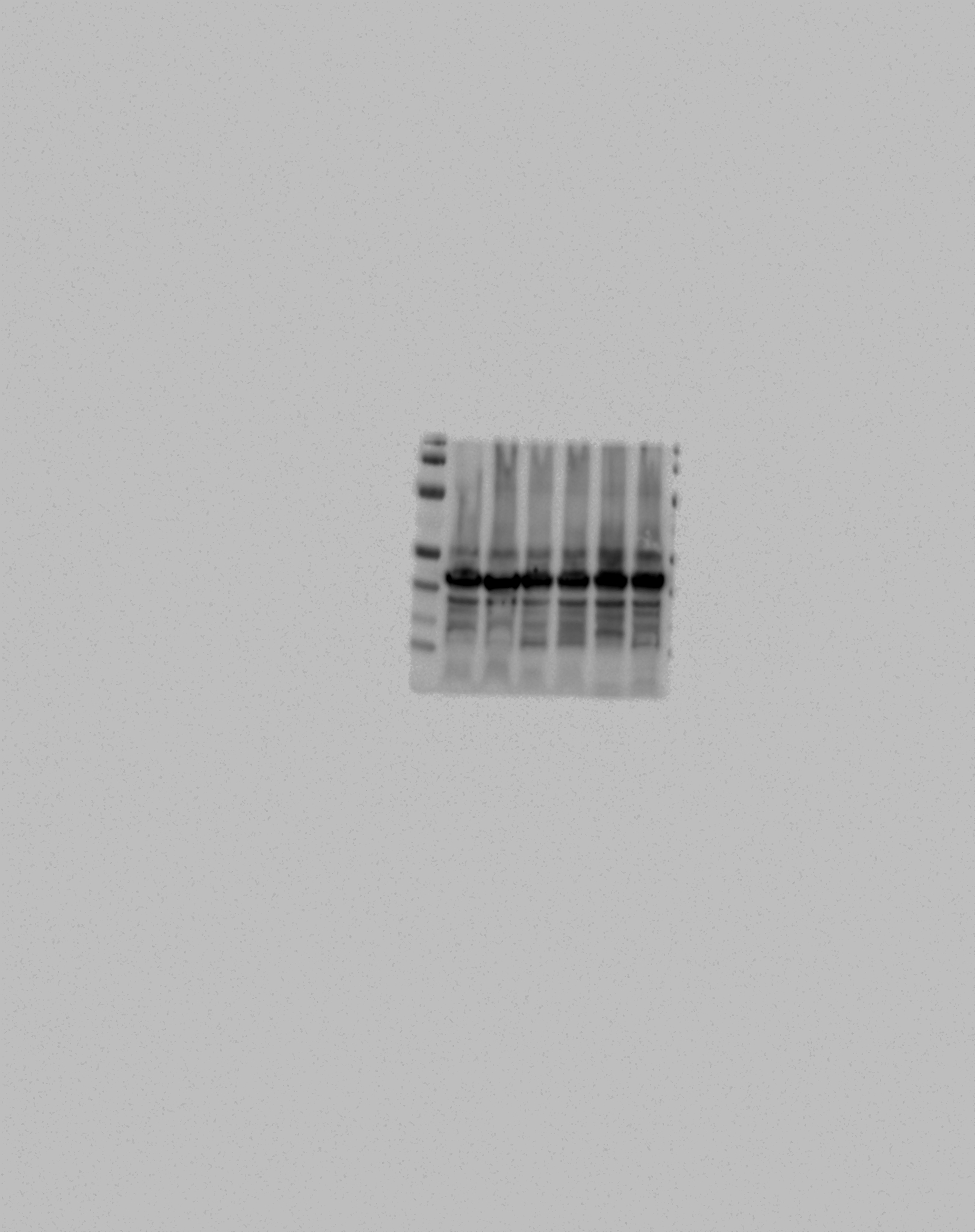

Supplement: Supplementary file 1 [file DataSheet1.zip › data/liver/4actin.jpg]

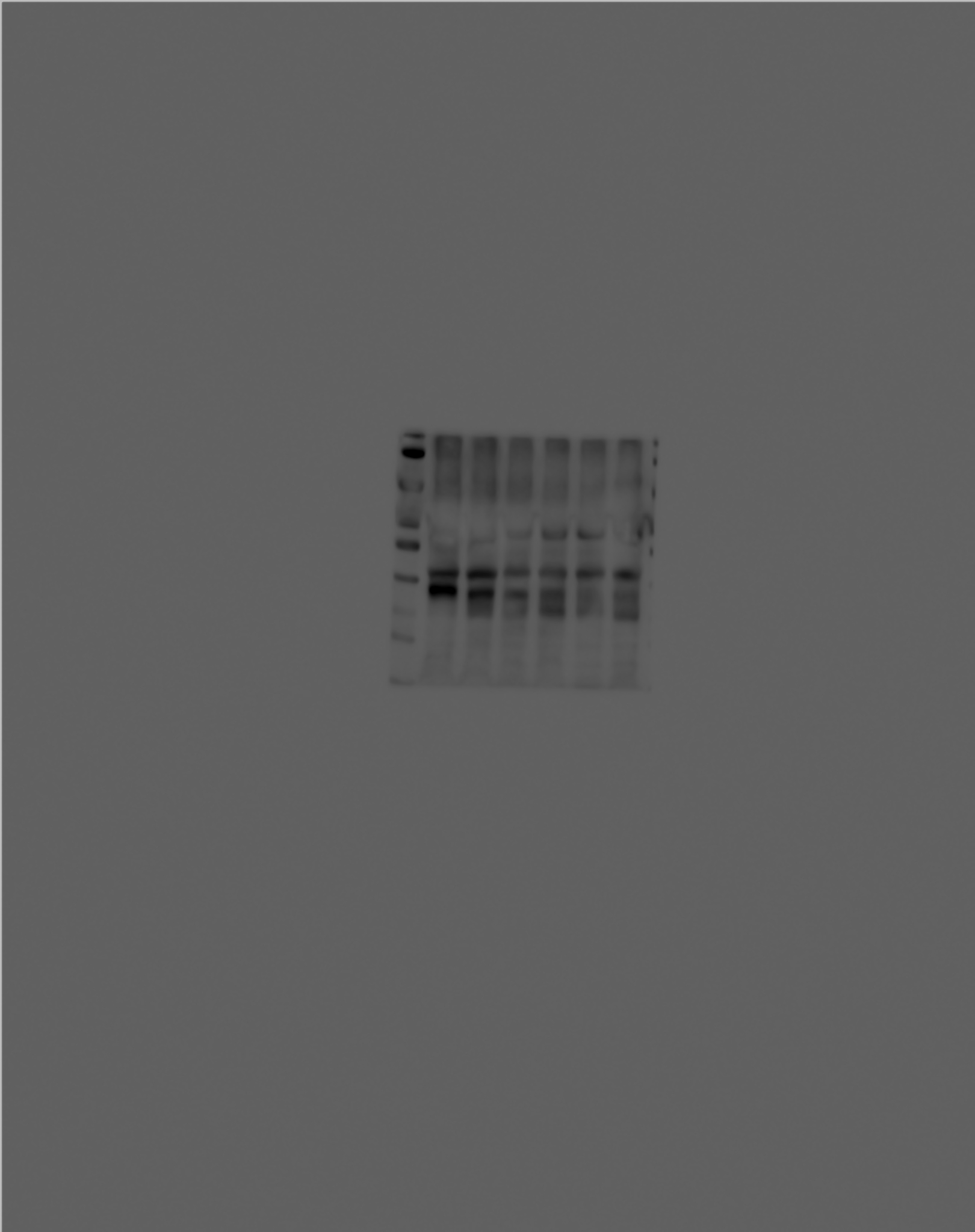

Supplement: Supplementary file 1 [file DataSheet1.zip › data/liver/4rdh10.jpg]

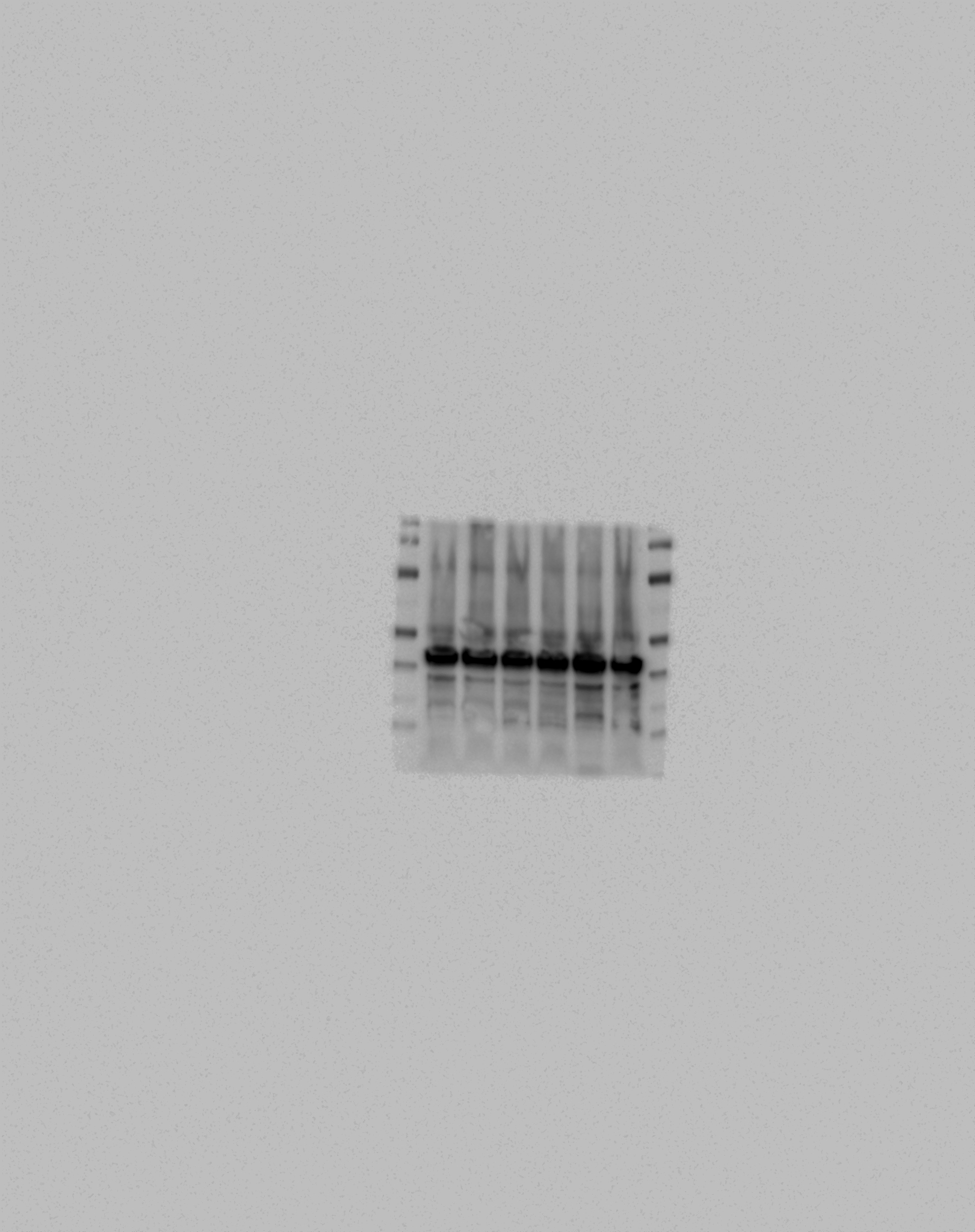

Supplement: Supplementary file 1 [file DataSheet1.zip › data/liver/5actin.jpg]

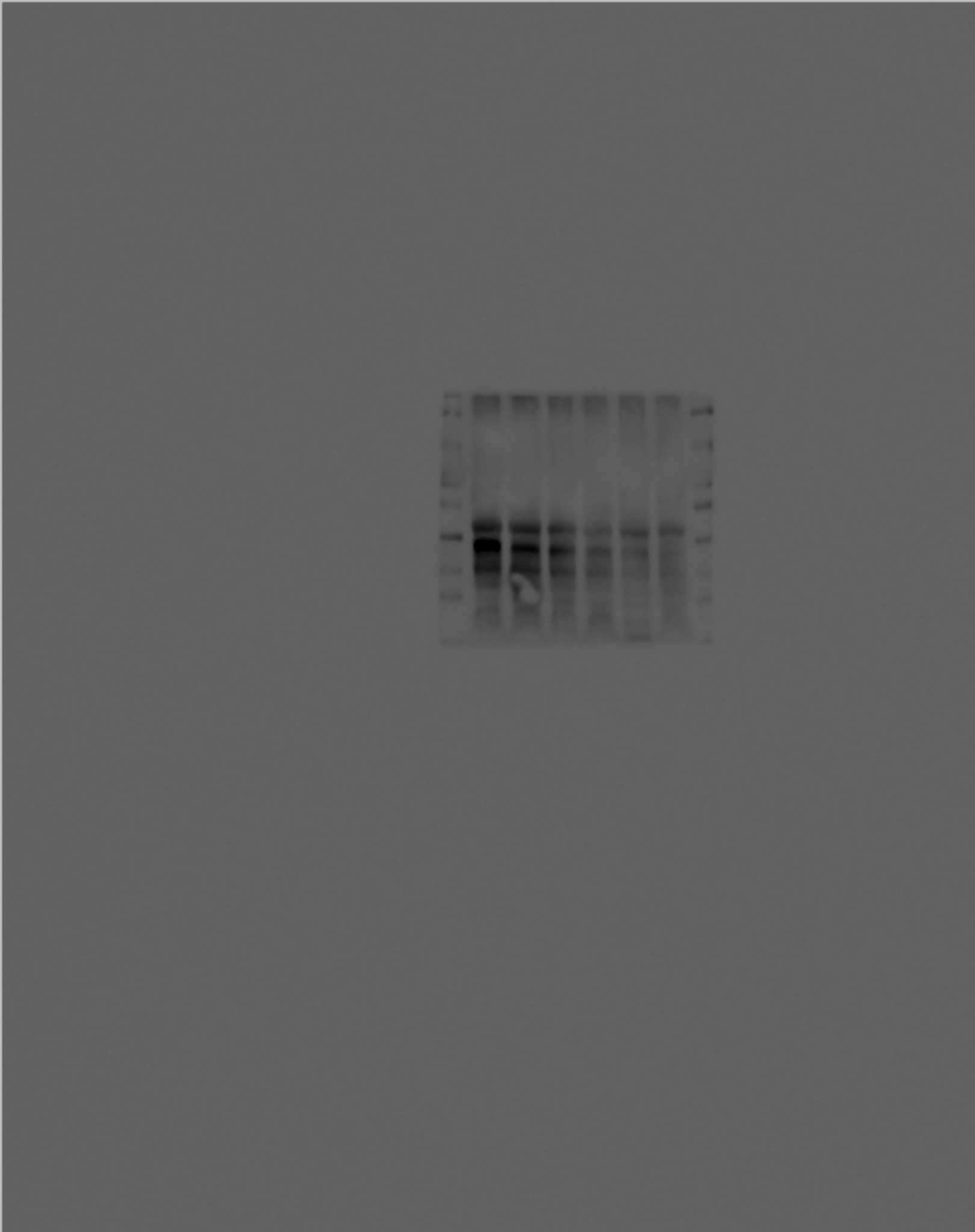

Supplement: Supplementary file 1 [file DataSheet1.zip › data/liver/5rdh10.jpg]

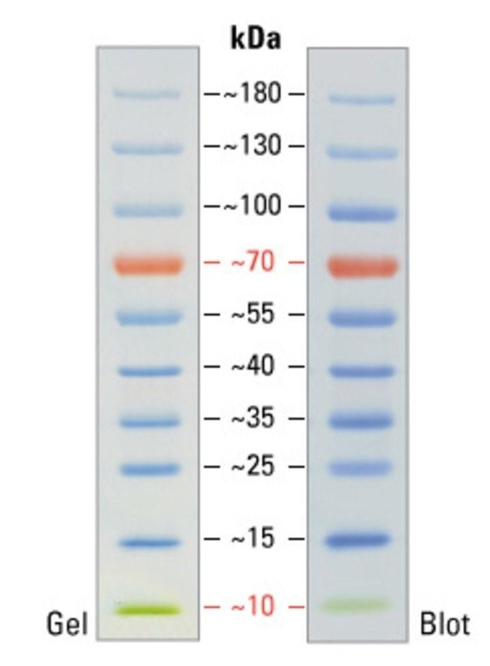

Supplement: Supplementary file 1 [file DataSheet1.zip › data/marker26616.jpg]
