# Supplementary material for: Identification of retinol dehydrogenase 10 as a shared biomarker for metabolic dysfunction-associated steatotic liver disease and type 2 diabetes mellitus
Source: Front Pharmacol. 2025 Jan 24;16:1521416. doi: 10.3389/fphar.2025.1521416 (PMC11802817; doi:10.3389/fphar.2025.1521416)
Supplement: Supplementary file 3 [file Image1.pdf]

We conducted an exploratory analysis of the normalized MASLD datasets (GSE33814 (green) and GSE48452 (yellow)) using principal component analysis (PCA) (Figure 1A). The results showed that the first two principal components (PC1 and PC2) explained 11.37% and 10.68% of the variance, respectively.The PC1-PC2 scatterplot showed a relatively even distribution of samples, and no clear grouping or clustering patterns were observed. The T2DM datasets (GSE76895 (green) and GSE89120 (yellow)) were merged and normalized with PCA. the first two principal components (PC1 and PC2) explained 15.45% and 8.87% of the variance, respectively. the PC1-PC2 scatterplot showed that the samples were relatively uniformly distributed, and no significant grouping or clustering was observed (Figure 1B). These data have been uploaded to the Supplementary Material.

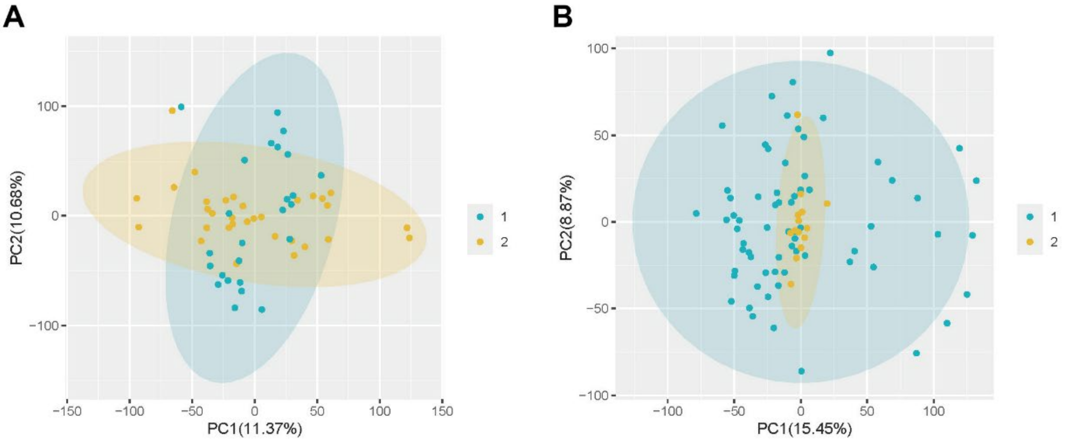

Figure 1. Principal Component Analysis (PCA) plots. MASLD datasets GSE33814 (green) and GSE48452 (yellow). T2DM datasets GSE76895 (green) and GSE89120 (yellow)
